# Disciplinary Proceedings as an Instrument for Breaking the Rule of Law in Poland

**DOI:** 10.1007/s40803-020-00146-y

**Published:** 2020-10-07

**Authors:** Katarzyna Gajda-Roszczynialska, Krystian Markiewicz

**Affiliations:** 1 dr. hab. assoc. prof. of Civil Procedure Department, Faculty of Law and Administration, University of Silesia, Katowice, Poland; 2 Prof. US, dr. hab. Civil Procedure Department, Faculty of Law and Administration, University of Silesia, Katowice, Poland

**Keywords:** The rule of law, Disciplinary proceedings, Independence of the courts, A right of access to the courts, Definition of the notion of a court, Reform of the justice system

## Abstract

This article advances the thesis that disciplinary proceedings may constitute a tool for breaking the rule of law in Poland. In 2017, as part of a package of legal changes to the judiciary, a disciplinary system was created in Poland to ensure that judges were subservient to the political will of the authorities. From the beginning, new disciplinary officers appointed by the Minister of Justice (the Prosecutor General) have targeted judges who disagree with unconstitutional changes to the judiciary. Disciplinary proceedings are by no means repressions that affect judges who demand that other authorities respect the rule of law in Poland. The article discusses, on a step by step basis, the practical mechanisms taken by the political authorities to break the rule of law in Poland. Particular attention is paid to the measures which have been taken concerning the judiciary. The article discusses the judgment of the CJEU on 19 November 2019 in combined cases C-585/18, C-624/18, and C-625/18 and the implementing resolution of the combined Civil, Criminal and Labour and Social Insurance Chambers of the Supreme Court on 23 January 2020 as well as the collapse of the rule of law in Poland from a practical perspective. The analysis of the recent events shows that after the so-called Muzzle Law (A bill amending the Act on the Organization of Ordinary Courts, the Act on the Supreme Court and the Act on the National Council of the Judiciary was submitted on 12 December 2019, and then voted on by the parliamentary majority in the lower house of the Polish Parliament (Sejm) on 20 December 2019.) came into force, the application of the resolution of the combined Civil, Criminal and Labour and Social Insurance Chambers of the Supreme Court on 23 January 2020 implementing the CJEU judgment in the joined cases C-585/18, C-624/18, and C-625/18 of 19 November 2019 can be and, in fact, is penalized by further disciplinary proceedings, which constitutes a real threat to the already weakened rule of law. Institutions and, above all, judges who are safeguarding the rule of law are being destroyed.

## Introduction

This article puts forward the notion that a proper form of disciplinary proceedings is a necessary element of independent and impartial courts. Independent and impartial courts in our opinion are active guardians of the rule of law. Furthermore, courts in democratic countries exercise judicial authority so that their main function is to ensure the right of access to a court under Art. 45(1) of the Constitution of the Republic of Poland or more widely under Art. 6(1) of the Convention for the Protection of Human Rights and Fundamental Freedoms^1^ or Art. 47 EU Charter of Fundamental Rights^2^ and S. 19(1) Treaty on European Union,^3^ a right which is only possible before an independent court. Guaranteeing individuals the right to have access to a court as a public subjective right towards the state is one of the basic concepts of a democratic country. Changes to disciplinary proceedings aimed at subordinating the courts to the executive authorities and leading to the freezing of the functioning of the courts constitute a tool for undermining the rule of law and, as a consequence, depriving parties of the right to have access to a court. In other words, recent experiences in Poland have shown that disciplinary proceedings form a means by which to interfere with the courts’ independence. Moreover, these changes are an element of dikastophobia—a dangerous strategy by the governing party aimed at preventing the executive and legislative powers being controlled by the judicial authority.^4^

## The Rule of Law and an Independent and Impartial Court System

The concept of the rule of law is certainly not new. The concept has a strong presence in legal theory,^5^ in traditions and branches of political theory^6^ and in sociology.^7^ Of course, the rule of law cannot be studied without taking historical and cultural contingencies into account.^8^ In Poland there are still strong demands for settling scores from the past. Many of these grievances involve real or alleged wrongs which are condemned today, and rightly so, but which often had legal authorization at the time or transgressed no existing positive laws when they were committed. Among the potential casualties of now applying the law to these past grievances are what are often said to be central elements of the rule of law such as: independent and impartial courts.^9^

In what follows we deal with the rule of law as a practical ideal. Needless to say, this maxim is not meant to be taken literally. Rather, the decisions made by legal actors—legislators, judges, and other officials—should be governed by certain ideals and standards.^10^ We share an opinion that the rule of law means “law plus standards”. From one point of view, narrowly conceived, the “classical” rule of law is a set of negative safeguards against an abuse of power. Officials are restricted by constitutional constraints, procedural rules, and institutional arrangements. Furthermore, such a conception is clearly supported by specific historical achievements and widely recognized principles of justice.^11^ A simple comparison between the elimination of the rule of law in communist times and the acceptance of only basic principles based on a positivist concept is not appropriate. Furthermore, a positivist conception^12^ is clearly unacceptable. The communist system collapsed over 30 years ago. There are no additional problems than those that existed in the 1990s, while completely new ones also appeared with a specific cultural background.

The rule of law will be operationalized by pointing to various institutional and extra-institutional aspects, taking into account the separation of powers. In recent years a new element has appeared in the discussion on the rule of law, the separation of powers^13^ and the role of independent judges. Judicial authority is exercised by courts and tribunals. Courts and tribunals would not be able to adjudicate without judges.^14^ An independent court is a court that is structurally, organizationally and functionally separate from other public authorities. Furthermore, a sovereign court is a court in which impartial judges adjudicate .^15^ A new paradigm of the court assumes the existence of a court equipped with jurisdictional authority as a guarantor for exercising the right to have access to a court, which is only possible before the court whose independence is guaranteed, irrespective of whether European law is or could be applied in a given case. It is consistent with the construct of the right to access a court constituting not only the right in itself but also a protective measure with respect to other subjective rights.^16^ The evolution of the CJEU’s opinions and the said approach adopted in the CJEU’s judgment of 27.02.2018 in the case C – 64/16 Associação Sindical dos Juízes Portugueses^17^ assume the creation of a multi-layered notion of the court. The judgment of the Court delivered in this case was as groundbreaking as it was surprising, even if the outcome itself was predictable.^18^ The political objectives of the Court’s decision are clear: the CJEU wanted to clarify that the organization of the national judiciaries is not exclusively a matter for each of the Member States separately, but that Member States are under an obligation, contained in primary EU law and supervised by the Court of Justice, to ensure that their courts and judges are independent ‘in the fields covered by EU law’.^19^ Independence is a “constitutional” value and should be verified at the EU and national level.^20^ The concept of independence^21^ must, on the one hand, fulfil European standards, while, on the other, it has to take into account the specific status of a judge as provided for by national legislation, so that making this criterion precise must always be an obligation for the national courts. The notion of independence includes internal and external aspects.^22^ The internal aspect of independence is nothing but the impartiality of the judge who should keep his/her distance from the parties to the dispute and their interests with respect to the subject matter of the dispute.^23^ The court should be objective and should not have any interest in settling the dispute in any specific way except by means of a strict application of the law.^24^ The external aspect of independence assumes that the adjudicating body is protected against any intervention and external pressure which risks the independence of its judgments.^25^ A necessary element of the external aspect is the correct formation of disciplinary proceedings so that they can fulfil their functions but not constitute a tool for exerting pressure on adjudicating bodies leading to their dependence on legislative and executive powers.

In this context it is beyond any doubt that one of the elements for ensuring the courts’ independence is a proper form of disciplinary proceedings. Furthermore, there can be no doubt that an improper form of disciplinary proceedings may create a tool for limiting the independence of judges. In other words, the CJEU transformed a case that initially seemed to be about the judicial review of an austerity measure into a decision on the organization of the European judicial system.^26^ That is why disciplinary proceedings should meet certain standards. This mechanism was used in the case of Commission v Poland (independence of the Supreme Court).^27^ Moreover, the CJEU has established its jurisdiction regarding the adoption of an interim measure as an instrument to ensure respect for the rule of law in Poland.^28^ The European Court of Justice’s order in Case C-791/19 R^29^ is the third time that the Court has granted interim measures that have been applied for by the Commission so as to preserve the rule of law in Poland .^30^

This judgment created a new dimension in the significant and active role of the courts in protecting the rule of law in the EU legal order. For a long time Article 2 TEU seemed to be nothing more than a mere proclamation of values with only limited implications (Article 7 TEU). It is generally argued that a violation of Article 2 TEU cannot itself be a ground for judicial action.^31^ Respect for the rule of law has always played an important role in the case law of the CJEU. References have been made to the principles of legality, legal certainty, the prohibition of arbitrariness in executive powers, the right to a fair trial before independent and impartial courts, the separation of powers and equality before the law. The cornerstone is a “Union based on the rule of law”. The strengthening of the EU’s core values with the Treaty of Lisbon and the rule of law backsliding in certain EU member states provided the context against which the Court is increasingly discovering the potential of Article 2 TEU, in conjunction with Article 19 (1) TEU, for ensuring respect for the rule of law in the EU legal order.^32^

Of course, the aspect of the separation of powers and the rule of law is very important, but we are in full agreement with Martin Krygier on the issue of the essence of the matter in question. What about institutional and extra-institutional aspects of the rule of law in Poland? Do we really have the rule of law in Poland? Does the law still really matter?

## The So-Called Reform of the Justice System in Poland (2015–2020)

Respect for the rule of law has always played an important role in the EU, particularly in the case law of the CJEU. References have been made to the principles of legality, legal certainty, the prohibition of arbitrariness in executive powers, the right to a fair trial before independent and impartial courts, the separation of powers and equality before the law.^33^ The cornerstone is a “Union based on the rule of law”. The member states should protect the structure and functioning of the EU legal order and especially the rule of law. On the other hand, in Poland in the last few years we can see a so-called “rule of law backsliding”. Rule of law backsliding has been defined by Pech and Schepple as “the process through which elected public authorities deliberately implement governmental blueprints which aim to systematically weaken, annihilate or capture internal checks on power with the view of dismantling the liberal democratic state and entrenching the long – term rule of the dominant party”.^34^ In Poland in 2020 the collapse of the rule of law continues.^35^ Since October 2015 the governing Law and Justice Party has been engaged in a so-called reform of the justice system in Poland.

The first safeguard that was eliminated by politicians was the *Constitutional Tribunal*. In transgressing the law as it stands (Art. 190 para. 2 of the Constitution of the Republic of Poland, henceforth the Polish Constitution), the rulings of the Tribunal in 2016 were not published until two years later (on 5 June 2018), when Poland thus made concessions in its dispute with the European Commission. This was done with a caveat that the rulings were issued in violation of the provision of the Act of 25 June 2015 on the Constitutional Tribunal, and that they were with reference to “a normative act that had lost its binding power”.^36^ The crisis was initiated in the autumn of 2015 and concerned the election of three judges of the Constitutional Tribunal by Parliament to the replace the judges already duly elected.^37^ The President refused to take the oath from those judges who had been properly elected,^38^ and they were therefore not allowed to take office. The *Sejm* (the lower chamber of the Polish Parliament) elected other judges in their place – referred to as stand-ins (doubles). They themselves said that they represented the government.^39^ Three other judges who had just been elected were not allowed to take office.^40^ The new president of the Constitutional Tribunal, Julia Przyłębska,^41^ and Mariusz Muszyński, the person whom she had appointed as her substitute with no justification, shuffled the panels of judges hearing cases according to their own liking.^42^ The new judges themselves examined the cases pertaining to the lawfulness of their own appointment, thereby completely ignoring the principle of *nemo iudex in causa sua*. The president of the Constitutional Tribunal expressed her own opinions on the constitutionality of the Act on the public television service. The Acts enacted in 2015 and 2016 regulate the Constitutional Tribunal in such a way that it violates the Constitution. This all resulted in constitutional bodies such as the Disciplinary Officer, the National Council of the Judiciary and the First President of the Supreme Court withdrawing their constitutional complaints to the Constitutional Tribunal as the Tribunal had become nothing more than a facade institution. At present, nobody in their right mind would see the Constitutional Tribunal as a guardian of the Constitution but rather as a highly politicised body.^43^ In 2016 prosecutors commenced proceedings concerning the Prime Minister’s refusal to publish verdicts of the Constitutional Tribunal. The prosecutors responsible for this case were changed on two occasions. Finally, the Minister of Justice achieved the desired result and the case was discontinued. In the decision published on 10 February 2017 the prosecutor argued that the Prime Minister – although he was obliged by the Constitution to publish all verdicts by the Constitutional Tribunal – may have had justified concerns that the publication of those rulings would be illegal. A former president of the Constitutional Tribunal, Marek Safjan, noted that this was the point when the rule of law came to an end in Poland.^44^ By early 2017 the Tribunal was firmly under the control of the ruling party (the Law and Justice (PiS) party). Over the past 5 years the Republic of Poland has been losing its internal state sovereignty.^45^

The government’s next plans were as follows: to target how judges and prosecutors are trained; how the ordinary courts operate; and how judges are promoted, disciplined and dismissed. The focus shifted to the National School of Judges and Prosecutors, the Polish National Council of the Judiciary (NCJ), the ordinary courts and finally the Supreme Court. All of these goals have been achieved. The laws that have been enacted and which amend the laws on the Supreme Court^46^ and the National Council of the Judiciary^47^ have introduced further mechanisms for destroying the autonomy of the courts,^48^ including extraordinary complaints, the new structure of the Supreme Court and last but not least, disciplinary proceedings.

A series of laws have been enacted, including as from January 2018 a law concerning the Polish Council for the Judiciary (KRS). The above-mentioned laws stipulate that it is the Sejm which will elect judges as members of the KRS. This is in violation of the constitutional principles of the independence and separateness of the judiciary. Taking into consideration the fundamental defectiveness of the proposed mechanism for appointing judges to the KRS, it does not matter whether Parliament elects by a simple or a qualified (three-fifths) majority. It should be stressed that the KRS is meant to ensure the independence of the courts and the impartiality of judges (Art. 186 Polish Constitution). The composition of the KRS cannot depend solely on the decision of the remaining two branches of power. This is why the Constitution clearly limits the number of KRS members elected by representatives of the executive (the President) and the legislative branch. The Minister of Justice (in Poland the Minister of Justice is also the Prosecutor General) is also a member of the Council.^49^ Such a solution is in line with Recommendation CM/Rec(2010)12 of the Committee of Ministers on the independence, responsibility and efficiency of judges which in para. 27 stipulates that “not less than half the members of such councils should be judges chosen by their peers.”^50^ Particular attention should be also paid to the fact that this law put a stop to the constitutional tenure of the KRS (Art. 6 Act on the National Council of the Judiciary) and introduced a joint four-year term in office for the members of the Council chosen from among judges (Art. 9a para. 1 Act on the National Council of the Judiciary). In the reasons for the bill, it was indicated that the criticised interim provisions respect the principle of the continuity of a constitutional body because the term of office of the present members of the Council shall expire on the date of the first day of the joint tenure of the new KRS members. In March 2018 the terms of all 15 serving judge-members were prematurely terminated, to be replaced by 15 judge-members chosen by parliamentary majority. In the end only 18 judges were even put forward for the NCJ as candidates. Since the amendment of the Law on the National Council of the Judiciary (NCJ) which entered into force in April 2018, 15 judge-members of the NCJ have been elected by parliamentary majority (previously they were elected by judges). In practice the number of professional and personal connections of present judge-members of the NCJ to the Ministry of Justice is so high that the newly-created body constitutes another body of the executive authority. If we add to that the 6 members elected by Parliament, the representative of the President and the representative of the Minister of Justice it turns out that 23 of the 25 members of the NCJ are going to be either elected or appointed by politicians. Therefore the KRS is in blatant violation of the rule to safeguard the independence of the Judiciary, to defend the Judiciary, as well as individual judges. The KRS undermines the application of EU law as to the independence of judges and tribunals, and thus also its effectiveness. For example: in a resolution of 23 January 2020 the Polish Supreme Court (the Grand Chamber of all the judges of three divisions) concluded that the Disciplinary Chamber did not satisfy the CJEU test of being an independent tribunal. It also decided that the KRS is not independent from the Executive. In a direct response to this judgement, the KRS has actively supported the disciplinary prosecution of judges who apply the CJEU test (see also the answer to question 9 in the letter of 13 March 2020). The first judgement in such a case has been delivered: a judge (Pawel Juszczyszyn) has been suspended from his judicial duties indefinitely. On 14 February 2020 further legislation was enacted in Poland. Under Article 107 of this law judges are liable to disciplinary procedures if they are adjudged to have engaged in political activity, such as protesting against the reforms, applying European Law as to the independence of judges and tribunals, and for submitting questions to the CJEU. The KRS is very much in favour of this law, and openly supports it. On 17 September 2018, an Extraordinary General Assembly of the ENCJ decided to suspend the membership of the KRS because it no longer meets the ENCJ requirement of being independent from the Executive and Legislature in a manner which ensures the independence of the Polish Judiciary (position paper added). Only the Polish Council for the Judiciary (KRS) voted against its own suspension. On 17 May 2020 the Board proposed to the General Assembly of the ENCJ, which will convene as soon as the corona crisis allows it to do so, that the Polish Council for the Judiciary (KRS) be expelled as a member of the network.

The politicians presently governing Poland have introduced changes which more or less amount to increased political oversight of the courts, with a significant limitation of the role of the professional self-governing corporation of judges. This is just as it was in communist Poland when the Ministry of Justice had special rights to dismiss judges without restriction by *shortening the terms of office of the presidents of the courts* or other functions, and to appoint people of their own choice in their place. All of the authority to appoint presidents of the courts at all levels has now been granted to the Minister of Justice, simultaneously stripping the organs of the professional self-governing bodies of the judiciary of any competences in this respect (Arts. 23–25 of the Act: Law on the organisation of the common courts^51^**)**. The new way of appointing the presidents of the courts is a departure from the standards as presented in the jurisprudence of the Constitutional Tribunal and a violation of the constitutional principle of the separateness and autonomy of the judicial authority (Art. 178 para. 1 Polish Constitution).^52^ As many as 130 court presidents have been removed from office thus far.^53^ They have been informed of the fact by fax or courier mail, and the notifications were dated before the time of their receipt. Usually they were sent either on a Friday afternoon or when the judges were on holiday and they only learnt of this from the media. Their replacements were often “clerk judges” from either the Ministry of Justice or other courts, in which they had not adjudicated. Moreover, there is now also the controlling authority granted to the Minister of Justice and his subordinates. The Ministry is to exercise oversight of the courts via “a supervisory service consisting of judges delegated to the Ministry of Justice pursuant to Art. 77.” There are over 160 of them, with the number continuously increasing as judges who are willing to cooperate with the authorities are rewarded with such an appointment. At the same time, “fast promotion tracks” have been introduced, i.e. an accelerated means of promotion leapfrogging over one tier of the courts, namely moving directly from the district courts to the courts of appeal (Art. 64 Law on the organisation of the common courts).^54^ Taking into consideration the career promotion procedure, and in particular the determining role of the National Council of the Judiciary, one can expect fast promotion tracks for candidates supported by the party presently in power, which can create a serious risk of the career promotion process becoming political. These fears are well founded as those who are presently supported by the Ministry have been delegated to adjudicate in courts two instances higher (judges from the district courts to the courts of appeal). This is an unprecedented situation.

The next step was a bill amending the Act on the Organization of the Ordinary Courts, the Act on the Supreme Court and the Act on the National Council of the Judiciary which was submitted on 12 December 2019, and then voted on by parliamentary majority in the lower house of the Polish Parliament (Sejm) on 20 December 2019. It gained the well-deserved nickname of being a ‘muzzle law’, as it seems to be the greatest ‘achievement’ of the executive and the parliamentary majority on the path leading to the political subordination of the polish judiciary. This new so-called muzzle law introduces new types of disciplinary torts for judges, politicizes new disciplinary proceedings against judges even more so, deprives the bodies of judicial self-government of any significance (e.g. they have lost the right to give opinions on candidates for the office of a judge and on candidates for senior judicial positions, as well as the right to adopt critical resolutions regarding changes to the justice administration), imposes an obligation on judges to disclose their affiliation to judicial associations (information on this will be posted on the Internet), gives the President the right to correct any defectiveness in the nomination procedure for a judge and enables the ruling party to take over the position of the First President of the Supreme Court by empowering each member of the General Assembly to nominate its own candidate, thereby reducing the quorum necessary for the election and enabling the President to nominate the temporary president of the Supreme Court for the election period.

After the subordination of the Public Prosecution Office, the Constitutional Court and the National Council of the Judiciary to the political authority, the Supreme Court became the last standing independent body for legal protection in Poland. Firstly, they have changed the method for the allocation of cases between judges of the Supreme Court. Contrary to the ordinary courts, where the cases are allocated at random by an electronic system, in the Supreme Court they are allocated by the President of the Court. The next change was the creation of two entirely new Chambers, the members of which are chosen by the new politicized National Council of the Judiciary (the only requirement to become a member is 10 years of experience in any legal profession): they are the Disciplinary Chamber and Chamber of Extraordinary Claims and Public Affairs. The President of the Supreme Court, Prof. Malgorzata Gersdorf, the last key institution in the judicial system not fully controlled by the PiS government, has been replaced by a person affiliated to the PiS. Professor Małgorzata Manowska was appointed judge of the Supreme Court in a procedure that was grossly in breach of the Constitution.^55^ The involvement of an incorrectly appointed National Council of the Judiciary (a breach of Article 179 in connection with Article 187, item 1 of the Constitution) and the absence of a countersignature by the Prime Minister on the announcement of the recruitment (a breach of Article 144, items 2 and 3 of the Constitution) has resulted in the ineffectiveness of the election and nomination application submitted to the President of the Republic of Poland. She was then appointed First President of the Supreme Court by means of votes from those who were also incorrectly appointed as judges of the Supreme Court, despite the lack of support from the majority of the members of the Supreme Court Assembly and the lack of an Assembly resolution as required by the Constitution (a breach of Article 183, item 3 of the Constitution), with the Assembly being chaired incorrectly and by unauthorized persons. As a result, this position was assumed by a person who was dependent on a representative of the executive authority.

Furthermore, the disciplinary proceedings were changed to limit the procedural guarantees of judges. What especially raises doubts is the change in appointing disciplinary officers. The Minister of Justice was given the arbitrary right to appoint the deputy disciplinary officer for judges of the common courts and his two deputies.^56^ This disciplinary officer chose deputy disciplinary officers at the courts of appeal and regional courts from among the candidates presented to him by the general assembly of judges.^57^ The Minister of Justice arbitrarily determined the number of disciplinary judges at the courts of appeal (S. 110 c of the Law on the Common Court System) and appointed the judges to be members of such courts. This appointment is binding (S. 82 c of the Law on the Common Court System). What is more, in the case of any disciplinary proceedings against any judge, the Minister of Justice may appoint his own ad hoc disciplinary officer (S. 112 b of the Law on the Common Court System), which excludes any previous officer and is equal to the instigation of proceedings. The prosecutor may also be a disciplinary officer, which enables “manual control” of all proceedings. This means that those who exercise executive authority can have a direct influence on who accuses and tries judges. Additionally, the Minister of Justice is entitled to lodge a binding objection against the decision of the disciplinary officer on a refusal to instigate disciplinary proceedings and against the decision on the discontinuance of proceedings.

Granting the Minister of Justice the right to lodge an objection leads to the situation where his position will be highly privileged with respect to other entities entitled to submit a request to instigate an investigation by the disciplinary officer. The said objection as indicated above is binding as far as the disciplinary officer is concerned, as well as the recommendations concerning further proceedings. This means that the disciplinary officer may be obliged to instigate or continue disciplinary proceedings even though there are no grounds for this in his opinion. What is more, this right is completely arbitrary, and the possibility of lodging an objection on an unlimited number of occasions in the same case may - in extreme cases - lead to a constant prolongation of a situation in which a given judge will be faced with disciplinary accusations. Such a solution has to be evaluated also in the context of another provision under which the disciplinary limitation period does not run during the disciplinary proceedings until the day when the final and binding decision ending disciplinary proceedings is taken. This means that the Justice Minister has gained the possibility of having a significant influence on the course of disciplinary proceedings, meaning that certain judges are left with permanent accusations against them. Such a solution not only undermines the independence of the judiciary but also contradicts the essence of the statute of limitations. It means that, in practice, with such a system of disciplinary proceedings, the Minister of Justice may not only instigate disciplinary proceedings against a specific judge but may also choose the disciplinary officers in each case, as well as personally appointing his own disciplinary officer to proceed against a given judge, or may leave him or her with Kafkaesque permanent accusations against him/her. He also has an influence on the appointment of disciplinary court members at first instance. The Minister of Justice may also challenge a decision to discontinue proceedings in cases in which he has submitted the petition for instigating the disciplinary proceedings and refer the case back to the disciplinary court. The clear conclusion is that this is an inquisitorial model of proceedings.

Attention should also be paid to the so-called “Panel for the Justice Minister’s measures taken in the disciplinary procedure against judges and assistant judges”, created at the Justice Ministry whose aim is to “carry out an analysis and present the Justice Minister’s recommendations” within the scope of the “disciplinary procedure against judges”. In practice, according to media reports the members of this panel have spread untrue and defamatory information about judges. As a result of the disclosure of the defamatory attacks^58^ the Panel, has been dissolved.

Lay judges have been added to the panels at the Supreme Court’s Disciplinary Chamber. These lay judges are elected by the Senate (the upper chamber of the Polish Parliament). This in effect means that disciplinary courts have become political. It should be underlined that active politicians will choose the lay judges so a social element decides on the disciplinary liability of judges, including their removal from office.

A separate issue also involves the Disciplinary Chamber of the Supreme Court,^59^ as the court at second instance and it may be the court at first instance in disciplinary proceedings against judges from the ordinary courts, e.g. if they are accused of committing a crime. This Chamber has many features of a special court which does not fit within the catalogue of the justice administration bodies mentioned in S. 175 of the Constitution of the Republic of Poland. This Chamber is a fully autonomous and separate unit which, besides its name and location, has nothing further in common with the Supreme Court but only operates under its auspices. It has a separate president whose status is so special in comparison to other presidents of other chambers that his rank is practically as high as the First President of the Supreme Court, and in certain aspects he even has wider competences (compare s. 20 of the Supreme Court Act in relation to S. 35 § 3 fourth sentence of the Act on the Supreme Court); he also has a separate budget and office.

The President of the Disciplinary Chamber not only occupies an autonomous position with respect to the First President of the Supreme Court but also has an influence on the administrative functioning of disciplinary courts at first instance. The President of the Disciplinary Chamber of the Supreme Court not only appoints the presidents of the disciplinary courts for a 3-year term of office but may also dismiss them during their term in office based on general and unspecific grounds such as “a gross or persistent failure to meet professional obligations” or if “the further performance of the function cannot be combined for other reasons with the administration of justice”. He also may inspect the operations of the disciplinary courts at first instance. The President of the Disciplinary Chamber of the Supreme Court determines, thereby arbitrarily ignoring the random choice provisions (S. 110§ 3 LCCS), the disciplinary court that is competent to hear the case at first instance and this holds true for all forms of disciplinary misconduct. The Disciplinary Chamber is comprised of persons appointed by the new National Council of the Judiciary which is considered to be a body that is dependent on the political authorities. Half of them are made up of former prosecutors, including a prosecutor who interrogated the suspect during an earlier stage of the proceedings^60^ and other persons affiliated with the political authorities. It should be added that despite the resolution on the joined Civil, Criminal and Labour and Social Insurance Chambers of the Supreme Court dated 23 January 2020 finding that the DC is not a court and its decisions have not been binding since it was formed, members of the National Council of the Judiciary voted in a secret ballot on 31 January 2020 to elect 6 candidates for the Disciplinary Chamber of the Supreme Court, including persons connected with the so-called defamatory attacks^61^ and those who are simultaneously members of NCJ (which in many legal systems would itself give rise to the special disciplinary liability of members of the National Council of the Judiciary). What is also specific about this Chamber is its ability to waive a judge’s immunity and, as mentioned above, to accuse judges of committing a crime. Both of these competences are now used for repressive purposes. In the context of the said regulations, we may state that there is no doubt that this body has been created in order to comply with the case law of CJEU and^62^ ECHR.^63^

In order to complement the disciplinary system so-called “Muzzle Law” was passed. This new so-called “Muzzle Law” has introduced new types of disciplinary torts for judges and it has politicized new disciplinary proceedings against judges even more so (e.g. decisions on waiving a judge’s immunity and the temporary detention of a judge can only be made by the Disciplinary Chamber of the Supreme Court, the establishment of which was in conflict with the Constitution and EU law). It has introduced further changes in S. 107, 109, 110, 112, 114a, 128 LCCS with respect to disciplinary proceedings. The changes contained therein with respect to disciplinary proceedings are aimed at the situation where Polish judges do not respect the decisions of the CJEU and SC, do not protest against a breach of the Constitution by the legislative and executive authorities and do not carry out their obligations to maintain law and order. The changes provide for new types of misconduct and constitute grounds for holding a judge accountable for disciplinary reasons, where unclear and sweeping criteria are used which do not meet the constitutional requirement of acts prohibited by the law having been determined. It is even more striking that, in principle, these forms of misconduct (which do not constitute a crime) result in the judge in question being removed from the profession or transferred to another court.

In particular, S. 107 § 1 (3) LCCS provides for the liability of a judge for “actions questioning the existence of the professional relation of the judge, the effectiveness of the appointment of the judge”, “or the empowerment of the constitutional body of the Republic of Poland”. It leads to the judges in question being prohibited from sitting on the “neo-NCJ”, exercising the prerogative of the President or being elected as representatives of the NCJ. The sanction provided in this case is dismissal from office. Next in S. 107 §(4) LCCS the Sejm has introduced a disciplinary tort consisting of engaging in a public activity which cannot be reconciled with the principles of the independence of the courts and the sovereignty of judges. The entry into force of this regulation means that, contrary to European standards, judges will not have a public right to oppose changes that affect law and order. The changes introduced in S. 110 of LCCS lead in fact to depriving the disciplinary courts of competences concerning the waiver of a judge’s immunity, permission to hold him criminally accountable and to arrest him temporarily and then to refer the case to the Disciplinary Chamber of the Supreme Court. So, the basic sanction for verifying the correctness of the way judges were appointed and their jurisdictional authority involves dismissal from office. It means that when applying European law Polish judges will be penalized with at least the loss of their job. Finally, the system is meant to create a situation where only judges who do not criticize other authorities, and do not question the correctness of the measures taken by the other two authorities, will be left in Poland.

What is especially dangerous is that the most important disciplinary cases will be heard in both instances by the same body (a special court) and in such a case the penalty will in principle be dismissal from office or a transfer to another court. Such a body cannot be objective, is certainly not independent from other authorities and is not sovereign if it adjudicates in cases in which it is a priori interested in a specific decision, due to the way it is formed and its structure.

The Act has changed the principles for appointing deputy disciplinary officers, entrusting this competence to the disciplinary officers of the common courts without indicating any criteria or requirements for obtaining permission, making the appointment decision completely arbitrary. Simultaneously the self-governing judiciary and judges have been deprived of having any influence on the choice of local officers. This means, above all, that a system has been created where officers will be appointed by the disciplinary officers of the common courts who, in turn, have been appointed by the Justice Minister who is a politician. This change will increase the influence of the political authorities on the system of the disciplinary liability of judges.

The already existing system which has been changed as a result of the so-called Muzzle Law does not meet any constitutional, EU or international standards.^64^ All of the provisions in question create a mechanism for (ab)using disciplinary proceedings in order to eliminate the independence of the judiciary. The political authorities decide on everything. In our opinion this system is a system of “organised injustice”, but a legal form still exists. As Martin Krygier rightly observes, the form is not enough. Are the institutions as described in the books really working? Is the legal machinery that is in operation sufficient to allow us to speak about the rule of law in action? Is the rule of law independent from other principles of social life?^65^

## Case Study

In 2017 a disciplinary system was established under the Minister of Justice, Zbigniew Ziobro, to ensure that judges were subservient to the political will of the government. The disciplinary officer Piotr Schab and his deputies, Przemysław W. Radzik and Michał Lasota, have targeted judges who oppose unconstitutional changes to the judiciary, a judge who wore a T-shirt with the slogan “Constitution”, judges who have submitted preliminary questions to the EU Court of Justice, and judges who have delivered a verdict that is not in line with the preferences of the political authorities. There are currently 34 judges suffering formal persecution in the form of disciplinary charges after having opposed recent legislative measures because they undermine the rule of law in Poland, or because they have applied EU Court of Justice rulings. The charges may lead to suspension, a 40% loss of salary, and ultimately dismissal. On 4 February 2020 Judge Paweł Juszczyszyn was suspended and deprived of 40% of his salary after being the first judge to implement the verdict of the EU Court of Justice (of 19 November 2019) establishing criteria for independent and impartial courts; 26 judges have suffered informal persecution in the form of an unjustified change of duties or a change to their place work, threats of disciplinary charges, anonymous public harassment by representatives of the legislative or executive authorities and there has been one case of a judge being criminally charged. Judge Igor Tuleya may still have his immunity waived and be tried under the so-called Muzzle Law established in February 2020, to be applied retroactively for his ruling on 18 December 2017 allowing the media to be present as he ordered an investigation into a Law and Justice Party vote in Parliament.^66^ The Minister of Justice willingly uses this competence, informing the public and the courts of this, also with the help of public media currently under the influence of the ruling party and through electronic and social media. Widely announced preferences and suggestions concerning harsher punishment or selecting specific purposes of justice administration policy are intentionally being used so that they have a chilling effect as far as the judiciary is concerned. The new Disciplinary Chamber of the Supreme Court ensures that such measures are being intensified. The current system of disciplinary proceedings already provides the executive authority - as practice shows - with the possibility to exert enormous pressure on judges, which poses a serious threat to the independence of the judiciary. These tools constitute a source for exerting undue influence in an area in which judges are supposed to be independent, and, as a result, it may even lead to making judgements “political”, as well as to the possibility of disciplinary sanctions depending on the content of such decisions. Such cases are prolific.

Let us take the case of Judge Paweł Juszczyszyn as an example.^67^ Judge Paweł Juszczyn is a District Court Judge in Olsztyn who, due to a decision by the Minister of Justice, was seconded to adjudicate in the Regional Court in Olsztyn where he heard appellate cases against decisions issued by the district courts. While hearing an appeal in one case^68^ Judge Paweł Juszczyszyn, while carrying out the statutory obligation to check whether the proceedings were valid and whether the adjudicating panel had been properly empanelled, thereby adhering to the judgment of the CJEU on 19 November 2019 in joined cases C-585/18, C-624/18, and C-625/18, decided to verify the legal status of the judge who had been appointed by the neo-NCJ and who had delivered the first-instance decision. For this purpose he asked the Secretary General of the Sejm Office to present the original or officially certified copies of documents submitted to the Sejm Office concerning candidates’ applications and the list of citizens and judges supporting candidates for members of the National Council of the Judiciary, as per the resolution of the Sejm of Poland dated 6.3.2018.^69^ Judge Paweł Juszczyn also asked the Manager of the Sejm Office to present the originals or officially certified copies of documents containing the declarations of citizens or judges who had withdrawn their support for such candidates. In accordance with the judge’s request the said documents should have been sent to the Regional Court in Olsztyn and attached to case file no. IX Ca 1302/19 upr within 1 week of the request being served subject to a fine in the case of an unjustified refusal to provide all the requested documents. The documents were to be used for assessing, inter alia, the legal status of the body fulfilling the function of the National Council of the Judiciary with respect to meeting the criteria indicated in the CJEU judgment dated 19.11.2019 in joined cases A.K. (C-585/18), CP (C-624/18) and DO (C-625/18). Therefore Judge Paweł Juszczyszyn, when requesting the Sejm Office for the said documents, was acting under and within the limits of the law by applying the CJEU judgement of 19.11.2019, which was an obligation. Paweł Juszczyszyn was the first Polish judge to take responsibility for enforcing this CJEU judgment and his decision was met with an immediate reaction .^70^ On 28.11.2019 the deputy disciplinary officer for judges of the common courts, Michał Lasota, instigated disciplinary proceedings against Judge Paweł Juszczyszyn, accusing him of committing the crime of an abuse of power. In the opinion of the deputy disciplinary officer Judge Paweł Juszczyszyn, when requesting these documents from the Sejm Office in order to verify the status of the new National Council of the Judiciary, had exceeded his competences by granting himself the authority to verify the correctness, including the legality, of the election of members of the National Council of the Judiciary, and, as a consequence, granting himself the authority to assess the decision of the President of the Republic of Poland on the appointment of the judge in question. The deputy disciplinary officer for judges of the common courts also accused Judge Paweł Juszczyszyn of presenting his own assessment of the situation to the media. Consequently, the disciplinary officer accused Judge Paweł Juszczyszyn of not telling the truth about the facts in the applications in question. In turn the president of the District Court in Olsztyn, Maciej Nawacki, and simultaneously a member of the body performing the function of the National Council of the Judiciary whose status was to be examined by the judge from Olsztyn, ordered Judge Paweł Juszczyszyn to take a temporary leave of absence. However, certain government media and those favouring the government started a media campaign aimed at presenting Judge Paweł Juszczyszyn in the worst possible light. On 5.12.2019 the Labour and Social Insurance Chamber of the Supreme Court found that the Disciplinary Chamber of the Supreme Court is not a court as defined in EU law, and is not a court within the meaning of national law. The Labour and Social Insurance Chamber also found that the current National Council of the Judiciary is not an impartial body, is not independent from the executive and legislative authorities, and that the interpretation contained in the CJEU judgement of 19.11.2019 is binding on every court in Poland and all the organs of state authority. So verifying the legal status of the judges with respect to the SC challenging the impartiality and independence of the National Council of the Judiciary from the legislative and executive authorities was indeed an obligation for the Regional Court in Olsztyn. Measures taken by the disciplinary officer for judges of the common courts were improper and were part of the general trend of prosecuting judges for the content of their judgments when these decisions are not convenient for the ruling party or when they adhere to European law, including CJEU judgements. The General Assembly of the Judges of Olsztyn Region fully supported Paweł Juszczyszyn in their resolution dated 2.12.2019, demanding inter alia his forthwith reinstatement in the position which he held, and condemned the measures taken by the political authority, the disciplinary officers and the president of the District Court in Olsztyn, thereby demanding the immediate dismissal of the disciplinary officers in question.^71^ On 4 February 2020 the Disciplinary Chamber sitting in the Supreme Court building and consisting of three persons took a decision to suspend Judge Paweł Juszczyn and to reduce his remuneration by 40% pending the disciplinary hearing. Therefore this three-person chamber changed the earlier decision of the Disciplinary Chamber with another composition which did not allow Judge Paweł Juszczyszyn to be suspended. Lawyers representing Judge Paweł Juszczyszyn, although they were physically present in the Supreme Court building on 4.02.2020, did not enter the courtroom as this would amount to legitimizing the disciplinary chamber and its members. Next Judge Maciej Nawacki, who is a member of the politicised body performing the role of National Council of the Judiciary (the neo-NCJ) and is simultaneously the President of the District Court in Olsztyn having been nominated by the Minister of Justice, declared that he would carry out the decision of the disciplinary chamber. On 5.02.2020 Maciej Nawacki, in the presence of reporters from the TVN TV network, signed an order under which he suspended the inflow of cases to Judge Paweł Juszczyszyn during the period of his suspension and he also blocked his access to the court’s IT systems, his electronic passes to the court building and areas except for the judge’s own room, as well as placing a ban on giving him keys to any courtrooms and on his entry to the court outside of his working hours. Cases currently being heard by Paweł Juszczyszyn were referred to other judges.

Judge Igor Tuleya has repeatedly spoken in public about the state of the rule of law in Poland and in his statements he has always boldly defended the independence of the courts, the independence of judges and the principle of a democratic state subject to the rule of law, thereby openly criticising the unconstitutional changes introduced in the area of justice by those currently in power. Judges regularly meet with citizens in meetings on the rule of law, judicial independence, the independence of judges, the principle of the democratic rule of law and human rights. Judge Igor Tuley is also the instigator of a preliminary question to the Court of Justice of the European Union concerning the compatibility of Polish law with European law. As in the case of Judge Ewa Maciejewska from Łódź, the deputy disciplinary prosecutor for judges of the common courts, Michał Lasota, called on Judge Igor Tuleya to make a written statement concerning possible “judicial excessiveness”. In the opinion of the disciplinary prosecutor, the Polish court’s request to the Court of Justice of the European Union for a preliminary ruling on the compatibility of Polish law in the area of justice with European Union law may constitute grounds for initiating disciplinary proceedings. Judge Igor Tuleya’s educational and civic activities and judicial activity as a judge were met with a systemic response from the disciplinary prosecutor.^72^

Judge Waldemar Żurek has also repeatedly spoken in public on the state of the rule of law in Poland, and in his statements he has always boldly defended the independence of the courts, the independence of judges and the principle of a democratic state subject to the rule of law, thereby openly criticising the unconstitutional changes introduced in the area of justice by those currently in power. In January 2018, Judge Waldemar Żurek was dismissed from his position as the spokesman of the Regional Court in Kraków with regard to civil cases. In July 2018, Judge Waldemar Żurek was transferred from the 2nd Civil Appeal Division to the 1st Civil Division (1st instance), which was criticized by “Themis” (the Association of Judges) and “Iustitia” (the Association of Polish Judges), which described this decision as being politically motivated harassment of this judge and as an attempt to intimidate judges who openly act against actions aimed at political subordination to justice. Judge Waldemar Żurek had taken part in meetings with citizens, where current changes concerning the justice system, including the independence of the courts judges, were discussed. Judge Waldemar Zurek’s activities were met by a reaction from the disciplinary officer.^73^

Another example is the situation of the Judges of the Court of Appeal in Katowice, Aleksandra Janas and Irena Piotrowska. Judges Aleksandra Janas and Irena Piotrowska adjudicate appeal cases at this Court of Appeal. While examining an appeal against the judgment of the Gliwice regional court in a divorce case, the judges decided to examine the legal status of the judge sitting in the court who had issued the decision at first instance. To this end, on 11 December 2019, judges Aleksandra Janas and Irena Piotrowska decided to submit a legal question to the Supreme Court. The judges requested an answer to the question of whether it could have been be considered as a duly composed court if the adjudicating judge had been appointed by the new, politicized National Council of the Judiciary. Deciding on the status of a judge issuing a ruling is crucial to resolving a case. If the composition of the adjudicating court is contrary to provisions of law, this results in the invalidity of the proceedings. The decision issued by judges Aleksandra Janas and Irena Piotrowska aimed to assess, among other things, the legal status of the body performing the function of the National Council of the Judiciary in terms of meeting the criteria set out in the judgment of the CJEU of 19 November 2019 in joint cases AK (C-585/18), CP (C-624/18) and DO (C-625/18). Thus, judges Aleksandra Janas and Irena Piotrowska, by submitting a legal question to the Supreme Court, had acted on the basis and within the limits of the law, thereby applying the judgment of the CJEU of 19 November 2019, which they were obliged to do so. Aleksandra Janas and Irena Piotrowska together with judge Paweł Juszczyszyn are all judges who have taken responsibility for the implementation of the CJEU judgment of 19 November 2019, and this was met with the immediate reaction of a closed disciplinary/clerical system created by the politicians in power in Poland, which has always had one goal – to take control of the courts.^74^ On 15 December 2019, the Deputy Disciplinary Officer for Judges of the Common Courts, Przemysław Radzik, initiated disciplinary proceedings against judges Aleksander Janas and Irena Piotrowska, accusing them of committing the crime of an abuse of power. According to the deputy disciplinary officer, judges Aleksandra Janas and Irena Piotrowska had exceeded their powers, granting themselves the competence to assess how the new National Council of the Judiciary works in the way that it selects some Justices and using repression as a method to take control of this body and how specific judges are appointed with the participation of the new National Council of the Judiciary. Przemysław Radzik, the deputy disciplinary officer, determined that the legal question submitted to the Supreme Court by judges Aleksandra Janas and Irena Piotrowska amounted to unlawful interference in the statutory manner of appointing judges to adjudication panels. Then, on 18 December 2019, Przemysław W. Radzik submitted applications to the Disciplinary Chamber operating at the Supreme Court for the suspension of judges Aleksandra Janas and Irena Piotrowska from their official duties with a 25–50% reduction in their remuneration for the duration of this suspension.^75^ Judges Aleksandra Janas and Irena Piotrowska not only had the right but also the obligation to examine the legal status of a judge who had been appointed with the participation of the new National Council of the Judiciary, which clearly follows from the judgment of the CJEU of 19 November 2019 regarding criteria for assessing the status of the Disciplinary Chamber and the National Council of the Judiciary, issued in joined cases AK (C-585/18), CP (C-624/18) and DO (C-625/18). On 5 December 2019 the Supreme Court’s Chamber of Labour and Social Security found that the Disciplinary Chamber of the Supreme Court is not a court within the meaning of EU law, and is thus not a court within the meaning of national law. In addition, the Chamber of Labour and Social Security stated that the current National Council of the Judiciary is not an impartial body that is independent from the executive and legislative authorities. Further, it indicated that the interpretation contained in the judgment of the CJEU of 19 November 2019 binds every court in Poland, as well as any state authority. Therefore, an investigation into the legal status of judges in connection with the Supreme Court challenging the impartiality and independence of the National Council of the Judiciary from the legislative and executive authorities was indeed the responsibility of the Regional Court in Olsztyn. However, the actions of the deputy disciplinary officer Przemysław Radzik are unacceptable and are part of the observed general trend for prosecuting judges for the content of their judgments when these judgments are inconvenient for those in power, as well as for applying European law, including respecting the judgments of the CJEU All of these examples demonstrate how, in practice, the changed model of disciplinary proceedings is already being used to break the rule of law. In turn, the application of these measures against judges Aleksandra Janas, Irena Piotrowska, Paweł Juszczyn, Waldemar Żurek and Igor Tuleya is an example of the instrumental use of law by the disciplinary officer in order to have a freezing effect on the judiciary. It is also a blatant example of an abuse of power by officers for political purposes.

## The CJEU Judgement of 19 November 2019 in Joined Cases C-585/18, C-624/18, and C-625/18

As a result of doubts which appeared with regard to disciplinary proceedings, applications for a preliminary ruling on their interpretation were submitted by the Supreme Court. In the application for a preliminary ruling on interpretation in the case C-585/18 and in the second and third applications in cases C-624/18 and C-625/18, the court wanted to determine whether Arts. 2 and 19(1) (par. 2) TEU, Art. 267 TFEU and Art. 47 of the Charter of Fundamental Rights should be interpreted in such a way that the chamber of the highest court of a member state, such as the Disciplinary Chamber, which is to adjudicate in matters that are subject to EU law, meets, with respect to the conditions on which it was established and on which its members were appointed, the requirements of independence and impartiality required on the basis of EU law. If this is not the case, the court asked whether the principle of EU primacy should be interpreted in such a way that it requires the court to waive the application of national provisions reserving the competence for hearing such cases to the said court chamber. The basic doubts concerned whether in the light of the national provisions concerning the formation of a specific body, such as the Disciplinary Chamber, and defining in particular the competence held by it, its composition and conditions and principles for appointing judges sitting in the chamber, as well as the conditions and principles on which they were appointed, such a body and its members meet the requirements of independence and impartiality which a court must meet under Art. 47 of the Charter of Fundamental Rights if it adjudicates in a dispute in which the individual alleges a breach of EU law, as in this case. In this respect the CJEU is some kind of supreme court, assuming that the national courts co-share adjudication with the CJEU, being peculiar “common courts with respect to CJEU”, due to the decentralization of the application of EU law. The national courts play a leading role in maintaining the rule of law in the EU in cooperation with the Court of Justice, acting under national laws but also under European law in order to ensure that individuals have the effective rights to which they are entitled.^76^ By adopting this concept the CJEU assumed that this issue should be finally settled by the national court, after it completes all of its necessary findings. In this respect the CJEU reiterated that Art. 267 TFEU does not authorize the CJ to apply EU laws in a given case, but only to adjudicate on the interpretation of treaties and acts adopted by EU institutions. In accordance with determined case law on courts’ cooperation the CJ may, on the basis of the information contained in the case files, provide the national court with guidance as to the interpretation of EU law which may be useful for it while assessing the consequences of a given provision of this law.^77^ The CJ makes its interpretation on the basis of European law, and on the basis of national law this interpretation is left to the referring court - the Supreme Court. In its decision the CJ has formulated clear criteria which should be taken into account while assessing whether a judicial body is a court and its members meet the criteria of independence and impartiality resulting from Art. 47 of the Charter of Fundamental Rights. Above all it was underlined that all the facts connected with the appointment of judges of the disciplinary chamber should be assessed. The fact that members of the Disciplinary Chamber are appointed by the President of Poland cannot mean that such members are dependent on the political authorities or raise doubts as to their impartiality, if after their appointment they are not under pressure and do not receive any instructions while performing their duties .^78^ The role of the NCJ is also important. Irrespective of the assessment of the circumstances in which the new judges of the Disciplinary Chamber were appointed and the role which the NCJ plays in this aspect, the court should also determine other factors characterizing this body in a more direct way, such as e.g. the scope of its competences and its systemic role.^79^ In this context the CJ paid attention to the fact that under S. 131 of the new Act on the Supreme Court the Disciplinary Chamber may only be comprised of newly appointed judges, with the exclusion of judges who have already served as Supreme Court judges. The CJ noted that although the Disciplinary Chamber was formed as a chamber of the Supreme Court, contrary to other chambers forming this court it seems to have an exceptionally high degree of autonomy within the framework of this court and this is mostly the result of S. 20 of the new Act on the Supreme Court. The CJ underlined that the conglomerate of these circumstances analysed jointly may lead to the conclusion that it is not a court within the meaning of Art. 47 of the Charter of Fundamental Rights, especially if the said examination concerning the NCJ revealed that this body lacked independence from the legislative and executive authorities. So the CJEU ordered the national court to carry out such an assessment, taking into account, if necessary, specific reasons or purposes which will be touched upon before this court in order to justify certain disputed measures, whether the concurrence of the factors mentioned in points 143–151 of this judgment and all other duly proved important facts about which the court will be informed may raise in the opinion of individuals justified doubts as to the independence of the Disciplinary Chamber from the external factors, in particular from the direct or indirect influence of the legislative and executive authorities, and its neutrality with respect to a conflict of interests, and accordingly lead to a lack of independence or impartiality, which could undermine the trust which the judiciary should have in the eyes of individuals in a democratic society. As a result the CJEU stated that if this court came to the conclusion that this is indeed the case, it would mean that such a body would not meet the requirements resulting from Art. 47 of the Charter of Fundamental Rights and Art. 9(1) of directive 2000/78, as it would not be an independent and impartial court within the meaning of these regulations.

## The Resolution of the Combined Civil, Criminal and Labour and Social Insurance Chambers of the Supreme Court Dated 23 January 2020, Case BSA I-4110-1/20

As a result of the CJEU judgment the following judgments of the Supreme Court were delivered: 5 December 2019,^80^ and (II PO 8/18, III PO 9/18). The SC also decided on specific individual cases forming the basis of applications for a preliminary ruling on interpretation. The Supreme Court, taking into account the standards and principles for assessing the independence of the body and the sovereignty of judges, determined that the NCJ and the Disciplinary Chamber of the SC were bodies that lacked independence and sovereignty. In interpreting the SC case law there was a discrepancy in the issues covered by the consequences of the CJEU judgment dated^81^ 19.11.2019. As a result of the discrepancy in the resolution by the combined Civil, Criminal and Labour and Social Insurance Chambers of the Supreme Court dated 23 January 2020, case file no. BSA I-4110-1/20, at the request of the First President of the Supreme Court dated 15 January 2020 on solving the discrepancy in the interpretation of the law in the case law of the Supreme Court, it adopted a resolution under which an improper composition of the court under S. 439 § 1(2) of CrPC or the composition of the court being in non-compliance with the provisions of the law as defined in S. 379(4) of the CivPC will also occur if the composition of the court includes a person appointed as a judge of the Supreme Court at the request of the National Council of the Judiciary in the manner defined in the provisions of the Act dated 8 December 2017 on a Change to the Act on the National Council of the Judiciary and Certain Other Acts (Dz.U. of 2018 item 3). In the opinion of the Supreme Court an improper composition as defined in S. 439 § 1 (2) of CrPC or the non-compliance of the court’s composition with the provisions of the law as defined in S. 379(4) CivPC also occurs if the court’s composition involves a person appointed to be a judge in a common or military court at the request of the National Council of the Judiciary as as defined by the provisions of the Act dated 8 December 2017 on a Change to the Act on the National Council of the Judiciary and Certain Other Acts (Dz.U. of 2018 item 3) and if the defects in the appointment process lead in certain circumstances to a breach of the standard of independence and impartiality as defined in S. 45(1) of the Constitution of the Republic of Poland and Art. 6(1) of the Convention on the Protection of Human Rights and Fundamental Freedoms. In the opinion of the Supreme Court the interpretation of S. 439 § 1(2) of the CrPC and S. 37(4) of the CivPC adopted in points 1 and 2 of this resolution does not apply to judgements issued by the courts before it was adopted and to judgements which are still pending under the Code of Criminal Procedure before a given composition of the court. Point 1 of this resolution applies to judgments issued with the participation of the judges of the Disciplinary Chamber formed at the Supreme Court under the Act dated 8 December 2017 on the Supreme Court (Dz.U. of 2018 item 5 as amended) irrespective of when they were issued.^82^ The resolution by the combined Civil, Criminal and Labour and Social Insurance Chambers of the Supreme Court dated 23 January 2020 was the result of the reaction to the preliminary ruling concerning interpretation delivered by the CJEU on 19 November 2019 in combined cases C-585/18, C-624/18, and C-625/18, where an interpretation of EU law concerning the standards of independence and sovereignty pertaining the bodies, as required by S. 47 of CFREU and Art. 19 TEU, was presented. Moreover, the interpretation by the Supreme Court has erga omnes effect across the EU, which means that not only the CJEU but nearly all the national bodies are obliged to loyally implement this judgement (Art. 4(3) TEU).^83^

To begin with, a few formal issues should be underlined. The issue to be determined was not the issue of presidential prerogatives, the issue of the legislative competence of the Supreme Court or the constitutional understanding of the principle of the separation of powers.^84^ The resolution does not constitute law and is not an act of law in a strict sense. As a consequence, there were no grounds for the Sejm Speaker to submit an application to settle the dispute concerning competence between the Supreme Court and the Polish Sejm and between the President of Poland and the Supreme Court (case file no. Kpt 1/20) as there is no real dispute as such and the decision is inadmissible so the proceedings as a whole should have been discontinued. In the opinion of the Supreme Court both the circumstances surrounding its submission and the content of the justification leave no doubt that in fact we are here dealing with an abuse of competence, and the application was submitted in bad faith, not to settle a real competence dispute, but only to prevent the Supreme Court from exercising the rights which were granted to it by the legislator.^85^ As a consequence, the Constitutional Tribunal could not suspend the proceedings in the context of adopting a resolution, which was done by way of the decision dated 22 January 2020, and also could not suspend the application of the resolution by the decision dated 28 January 2020 on the suspension of the application of the resolution of the Supreme Court dated 23 January by the time the alleged competence dispute was heard by the CT (case Kpt 1/20). In this context we share the opinion that the application of the competence regulations with the purpose of controlling the resolutions of the SC is unlawful as it constitutes an abuse of control over constitutionality.

The resolution has a significant meaning, however, because, as a legal principle, it is binding on the adjudicating panels of the Supreme Court. Above all it is aimed at the harmonization of case law and to dispel all doubts concerning adjudication, and what is more significant, it aims to provide stability for the legal order. The resolution is balanced and is assumed to have long-term consequences.^86^ Its most significant element and general value is the finding that defectiveness in the proceedings leading to the appointment of a judge translates also into the attributes of the court as a body administering justice. It means that the lack of a guarantee of independence and sovereignty relating to the body prevents its jurisdictional function despite maintaining the formal status of a court. This is the result of the court being so defective that it cannot fulfil its jurisdictional function as it does not guarantee the right to access a court and the protection of all the other substantive rights. This is all the more so in light of the revealed support lists for the National Council of the Judiciary, which prove that the body was incorrectly formed and that the defectiveness in the procedure for the appointment of judges, and its structural character, leads to the situation where this body cannot properly perform its constitutional functions. The resolution differentiates the consequences of this defectiveness with respect to the disciplinary chamber and common court judges, making an assumption that the scale and scope of the procedural consequences of the defectiveness in the appointment of judges depending on the type of court in which the appointment takes place and its place in the structure of the judicial bodies are different. What is significant here is the status of Supreme Court judges, including Disciplinary Chamber judges who are only made up of judges appointed for the first time by the challenged National Council of the Judiciary and also the fact that the Chamber consists of judges nominated exclusively by the challenged NCJ. In the opinion of the Supreme Court, appointments to the Disciplinary Chamber are not appointments as judges as the Disciplinary Chamber is not a court as provided for by S. 175(1) of the Constitution and, as an exceptional court, it is prohibited as a result of the clear wording of S. 175(2) of the Constitution. It has also held that decisions of this chamber do not amount to court judgements as defined in S. 42(3) of the Constitution, and this provision, bearing in mind the presumption of innocence, applies also with respect to disciplinary liability. Judges cannot function without being assigned to a court. As a consequence, the Disciplinary Chamber is also not an European court within the meaning of CJEU case law. The presumption of innocence may be exclusively rebutted on the basis of a court judgement - but only by a court which satisfies the constitutional features of such a body.^87^ An exceptional court (a court not included in the list in S. 175(1) of the Constitution) during a time of peace cannot be a court within the meaning of the Constitution, as this would be contrary to the principle of legality. As a consequence, it may not deliver judgements in the name of the Republic of Poland. The formation of the Disciplinary Chamber has led to the exclusion of many individual cases from the competences of courts as in these cases the possibility of lodging an appeal to the court has been excluded. Due to the character of the disciplinary decisions it is a clear breach of S. 77(2) of the Constitution under which the law cannot preclude individuals from taking legal action to enforce breached freedoms or rights. If at first instance the case was heard by the courts hearing constitutional matters (this relates in particular to disciplinary cases relating to judges), the exclusive competence of the Disciplinary Chamber to hear appellate measures means that in such cases the constitutional principle of two-instance appellate proceedings no longer applies (S. 176(1) of the Constitution).^88^ Currently we have to deal with the fact that in many disciplinary cases the right of access to a court has been limited and the resolution of the Supreme Court removes this defect from the system. The Supreme Court, as the guarantor of the rule of law, has ensured the elimination of proceedings which were conducted in breach of Art. 47 of the Charter of Fundamental Rights, Art. 6 of the Convention as well as S. 45 of the Constitution of the RP. Further actions by the legislative and executive authorities indicate that this state of affairs has not been accepted and actions contrary to the judgment of the Supreme Court and the instigation of disciplinary proceedings in their current form must be evaluated within the context of the rule of law.

## Conclusions

So, do we have a rule of law in Poland? In our opinion Martin Krygier^89^ would not consider the current Polish legal system as one that satisfies any meaningful (or even formal) definition of the rule of law Three separate issues are important here.^90^

The first question is that of the efficacy of the law. The inefficiency of the machinery of justice is a great obstacle to establishing a State of Law in Poland.^91^ In recent years the functioning of the common courts has been systematically deteriorating. Reforms aiming to improve the functioning of the courts have been not carried out, and the COVID-19 pandemic has significantly worsened this situation. The present situation is critical.

Secondly, the rule of law is linked with Polish democracy and its fate, and so major threats to the rule of law amount to threats to Polish democracy in general.^92^ Nowadays, we have the dark scenario where the political class have turned into a democratically elected oligarchy. The problem is that we need to distinguish the rule of law from the welfare state, where privileged groups have a better social status. This better financial and social status has led to a situation in which people who considered themselves to be excluded are starting to support the populists. The populists created the authoritarian clientelism in Poland after 2015.^93^ That is why citizens and the authorities are “living with lies”.^94^

Thirdly, as the courts are the guardians of the rule of law, there is no rule of law without the right of access to a court. After the destruction of the institutions, the time has come to eliminate the judges, who are unfavourable to the populists. Disciplinary proceedings may create an instrument for breaking the rule of law in Poland. In 2017, as part of a package of legal changes to the judiciary, a disciplinary system was created in Poland to ensure that judges are subservient to the political will of the authorities. New disciplinary officers appointed by the Minister of Justice have targeted judges who oppose unconstitutional changes within the judiciary. Disciplinary proceedings are by no means the only forms of repression that affect judges who demand that other authorities respect the rule of law in Poland. The ongoing large-scale use of disciplinary proceedings against Polish judges who apply EU law will inevitably lead to a chilling effect and weaken the effectiveness of the law in the legal order. The idea of the EU as the community of law will be compromised. As no Polish legislation may prevent judges from other Member States applying the CJEU ruling of 19 November 2019, legal chaos undermining the stability of the EU legal order is an inevitable scenario. The system of EU law based on mutual trust and cooperation between the courts from all the Member States will be under a severe threat. Despite the judgment of the Supreme Court, the Disciplinary Chamber is still functioning. An analysis of the recent events shows that the application of the resolution by the combined Civil, Criminal and Labour and Social Insurance Chambers of the Supreme Court dated 23 January 2020 implementing the CJEU judgment in joined cases dated 19 November 2019 (C-585/18, C-624/18, C-625/18) can be and is penalized by further disciplinary proceedings, which constitutes a real threat to the already weakened rule of law.

The situation of the rule of law in Poland has been dramatically aggravated over the last few months. The Polish government has refused to respect the recent judgment by the CJEU regarding judicial independence in Poland (from November 19, 2019) and has enacted a law aimed at preventing its implementation. Polish judges already face disciplinary and criminal charges for submitting preliminary questions to the CJEU and for following its recent judgment. The Commission filed a complaint in October 2019, and in January 2020 – being especially concerned about the “Muzzle Law” before the Polish Parliament, which further tightened the disciplinary liability system – it applied for interim measures. On 8 April 2020, as a response to this complaint by the European Commission, the CJUE ruled that: “The Republic of Poland shall be required, immediately and pending the judgment in Case C 791/19, to suspend the application of the provisions of Article 3(5), Article 27 and Article 73(1) of the Supreme Court Act of 8 December 2017 (OJ L 2018, item 5), as amended, which constitute the basis for the jurisdiction of the Disciplinary Chamber of the Supreme Court in both the first and second instance, in disciplinary cases of judges; refrain from referring cases pending before the Disciplinary Chamber of the Supreme Court for consideration by a panel which does not meet the requirements for independence indicated in particular in the judgment of 19 November 2019, A.K. and others (Independence of the Disciplinary Chamber of the Supreme Court) (C 585/18, C 624/18 and C 625/18).” A new chapter in the conflict concerning the rule of law has begun: The PiS has openly rejected these Commission and CJEU decisions and appeals. What is at stake is no longer the conformity of the Polish judicial system with European standards but also respect for legally binding decisions by EU institutions. Most importantly, on 9 June 2020 in Warsaw, the meeting of the Disciplinary Chamber on waiving judge Igor Tuleya’s immunity took place against the decision of the CJEU. The Supreme Court Disciplinary Chamber has not yet revoked Judge Igor Tuleya’s immunity,^95^ but Poland has “crossed the rubicon” with its first political trial of a judge, in violation of an EU court injunction.^96^ The prosecutor's office appealed against this. The three person panel of Disciplinary Chamber will examine this appeal on 5 October 2020 in Warsow. The rise of populism in Poland is a real threat to democracy in Europe. If the populists will be able to effectively remove judges using disciplinary actions, the rule of law in Poland will be destroyed. The populists will then have realized their strategy to prevent judges from controlling government power.

## Footnotes

1 Dz. U. of 1993 no. 61, Item 284 as amended.

2 Dz. U. of 2009 no. 203, item 1569, hereinafter called – CFREU.

3 OJ of EU.C.07.306.1 as amended and Dz. U. of 2004 no. 90, item 864/30 as amended hereinafter called - TEU.

4 M. de Werd, ‘*Dikastophobia*’ *- a dangerous populist strategy to prevent judges from controlling government power*, https://europeancourts.blogspot.com/2020/02/dikastophobia-dangerous-populist.html.

5 For a formal conception of the rule of law see: Raz (1977), p. 195 at 196; Raz (1979); l Dicey (1959); Allan (1993); Arthurs (1985); Marshall (1971), p. 137; Unger (1976), pp. 176–181, 192–223. For a substantive conception of the rule of law see: Dworkin (1986); Dworkin (1985), pp. 11–16; Laws (1993), p. 59; Laws (1995), p. 72; Laws (1996), p. 622; Bellamy (1995); Sandel (1984); Mulhall and Swift (1992); Irvine (1996), p. 636 at 637; Dworkin (1977); J. Bell, Policy Arguments in Judicial Decisions. See also Craig (2016), pp. 467–487.

6 See: Markowski (2019), pp. 111–132.

7 See: Krygier and Martin (2008).

8 Selznick (2016), pp. 21–33.

9 Krygier and Czarnota (2016), pp. 2–3.

10 Selznick (2016), pp. 21–33.

11 Selznick (2016), pp. 21–33.

12 See: Morawski (2016), pp. 39 – 54.

13 See: Bellamy (1996), pp. 436–456.

14 *K. Gajda-Roszczynialska,* A test of six conditions of the EU standard of the notion of “court” and the Polish administration of justice – deliberations on the basis of the judgment of Court of Justice dated 27.2. 2018 in the case of C-64/16 Associação Sindical dos Juízes Portugueses. Iustitia 2018, no. 1, http://www.kwartalnikiustitia.pl/wp-content/uploads/2018/10/KGR-A-test-of-six-conditions-of-the-EU-standard-of-the-notion-of-court.pdf*A. Dąbrowski*, Ustrojowa pozycja sędziego, “Krajowa Rada Sądownictwa” No. 1/2014, p. 8.

15 As in: *K. Gajda-Roszczynialska*, A test of six conditions of the EU standard of the notion of “court” and the Polish administration of justice – deliberations on the basis of the judgment of Court of Justice dated 27.2. 2018 in the case of C-64/16 Associação Sindical dos Juízes Portugueses. Iustitia 2018, no. 1, http://www.kwartalnikiustitia.pl/wp-content/uploads/2018/10/KGR-A-test-of-six-conditions-of-the-EU-standard-of-the-notion-of-court.pdf.

16 The resolution of the joined Chambers: Civil, Criminal and Labour and Social Insurance Chamber of the Supreme Court dated 23 January 2020, Case BSA I-4110-1/20, point 13, http://www.sn.pl/aktualnosci/SiteAssets/Lists/Wydarzenia/AllItems/BSA%20I-4110-1_20_English.pdf.

17 Compare the discussion concerning this decision: Krajewski (2018), p. 395 et seq.; J. Barcik, Czy etos trzeciej władzy narusza jej więzi z Państwem Polskim?, Monitor Konstytucyjny: http://monitorkonstytucyjny.eu/archiwa/3461 M. Taborowski, A breakthrough judgment: CJEU finds that is has the right to assess the condition of administration of justice in EU countries https://archiwumosiatynskiego.pl/wpis-w-debacie/przelomowy-wyrok-trybunal-sprawiedliwosci-ue-uznaje-ze-prawo-oceniac-stan-wymiaru-sprawiedliwosci-panstwach-ue/; P.M. Van den Eijnden, AB 2018/220 - HvJ EU 27 February 2018, C-64/16, Associação Sindical dos Juízes Portugueses/Tribunal de Contas - Noot, Administratiefrechtelijke beslissingen; Rechtspraak bestuursrecht 2018 nº 26 pp. 1387–1390; L. Coutron, Observations à l'arrêt du 27 février 2018, Associação Sindical dos Juízes Portugueses contre Tribunal de Contas, affaire C-64/16, Jurisprudence de la CJUE 2018. Décisions et commentaires 2019, pp. 237–246; Coutron (2018), pp. 1417–1434; Simon (2018), pp. 11–12; Miglio (2018), pp. 421–431; Barcik (2018), pp. 23–29; García-Valdecasas Dorrego (2019), pp. 75–96; Miglio (2018), pp. 421–431; Pech and Platon (2018), pp. 1827–1854.

18 Bonelli and Claes (2018), p. 622.

19 Bonelli and Claes (2018), p. 623.

20 See: Coutron (2019), pp. 1417–1434; Simon (2018), pp. 11–12; Miglio (2018), pp. 421–431; Barcik (2018), pp. 23–29; García-Valdecasas Dorrego (2019), pp. 75–96; Miglio (2018), pp. 421–43.

21 See: K. Gajda – Roszczynialska, A test of six conditions of the EU standard of the notion of “court” and the Polish administration of justice – deliberations on the basis of the judgment of Court of Justice dated 27.2. 2018 in the case of C-64/16 Associação Sindical dos Juízes Portugueses. Iustitia 2018, no. 1, http://www.kwartalnikiustitia.pl/wp-content/uploads/2018/10/KGR-A-test-of-six-conditions-of-the-EU-standard-of-the-notion-of-court.pdf Compare in the aspect of the meaning of independence: Mokry (1985), p. 217. See also: Kosař (2017), pp. 96–123; Kosař (2016), pp. 23–142.

22 As in the judgement of the CJ on 16.2.2017 in the case of C-503/15, OJ of EU C dated 2017 no. 126, point 71; dated 19.9.2006 in the case of C 506/04, OJ EU C of 2006 no. 587, point 50.

23 Compare the judgment of the CJ on 16.2.2017 in the case of C-503/15, OJ of EU C dated 2017 no. 126, point 72; dated 19.9.2006 in the case of C-506/04, OJ EU C of 2006 no. 587, point 52; dated 22.12.2010 in the case of C-517/09, OJ EU C of 2010 no. 821, point 40; similarly the judgment of the CJ on 6.7.2000 in the case of C-407/98, OJ EU C of 2000 no. 367, points 34–37; decision of CJ dated 14.5.2008 in the case of C-109/07, OJ EU C of 2008 no. 274, point 24.

24 As in: K. Gajda – Roszczynialska, A test of six conditions of the EU standard of the notion of “court” and the Polish administration of justice – deliberations on the basis of the judgment of Court of Justice dated 27.2. 2018 in the case of C-64/16 Associação Sindical dos Juízes Portugueses. Iustitia 2018, no. 1, http://www.kwartalnikiustitia.pl/wp-content/uploads/2018/10/KGR-A-test-of-six-conditions-of-the-EU-standard-of-the-notion-of-court.pdf. Compare the judgments of the CJ dated 19.9.2006 in the case of C506/04, OJ EU C of 2006 no. 587, point 52; dated 6.6.2000 in the case of C-407/98, OJ of EU C dated 2000 no. 367, point 32.

25 As in: K. Gajda – Roszczynialska, A test of six conditions of the EU standard of the notion of “court” and the Polish administration of justice – deliberations on the basis of the judgment of Court of Justice dated 27.2. 2018 in the case of C-64/16 Associação Sindical dos Juízes Portugueses. Iustitia 2018, no. 1, http://www.kwartalnikiustitia.pl/wp-content/uploads/2018/10/KGR-A-test-of-six-conditions-of-the-EU-standard-of-the-notion-of-court.pdf Compare the CJ judgments dated 16.2.2017 in the case of C-503/15, OJ of EU C dated 2017 no. 126, point 74; dated 19.9.2006 in the case of C-506/04, OJ EU C of 2006 no. 587, point 50 and 51; dated 22.12.2010 in the case of C-517/09, OJ EU C of 2010 no. 821, points 39; decision of CJ dated 14.5.2008 in the case of C-109/07, OJ EU C of 2008 no. 274, point 23; dated 6.6.2000 in the case of C-407/98, OJ EU C of 2000 no. 367, point 34. See the similar judgements of the CJ dated 4.2.1999 in the case of C-103/97. p. I-551, point 21; dated 6.7.2000 in the case of C-407/98 p. I-5539, point 36; see also the judgement of ECHR dated 28.6.1984 in the case *Campbell and Fell* versus *United Kingdom*, A series no. 80, §78.

26 Bonelli and Claes (2018), p. 623.

27 ECJ 24 June 2019, Case C – 619/18 Commission v Poland (independence of the Supreme Court), EU:C:2019:531. See: Kmieciak (2019), pp. 143–150; Filipek (2019), pp. 4–14; Taborowski (2019), pp. 15–25; Świątkowski (2019), pp. 7–22.

28 See: ECJ 20 November 2017, Case 441/17 R, Commission v Poland (Puszcza Białowieska) EU:C:2017:877; ECJ 24 June 2019, Case C – 619/18 Commission v Poland (independence of the Supreme Court), EU:C:2019:531.

29 See ECJ 8 April 2020, European Commission v Republic of Poland., Case C-791/19 R. EU:C:2020:277.

30 See: L. Pech, Protecting Polish Judges from the Ruling Party’s “Star Chamber” The Court of Justice’s interim relief order in Commission v Poland (Case C-791/19 R), 9 Apr 2020; https://verfassungsblog.de/protecting-polish-judges-from-the-ruling-partys-star-chamber/.

31 Kochenov (2013), p. 148. 2 Kochenov and Pech (2015), p. 520; Müller (2015), p. 141 at p. 145.

32 Van Elsuwege and Gremmelprez (2020), p. 31.

33 Van Elsuwege and Gremmelprez (2020), p. 25–26.

34 See: Pech and Scheppele (2017), p. 3 at. p. 10.

35 See: Wyrzykowski (2019), p. 417–420.

36 It concerned judgments of the Constitutional Tribunal declaring that the Acts amending the law on the Constitutional Tribunal were unconstitutional. The Constitutional Tribunal’s judgment of 9 March 2016 stipulated that the amendment of the Act on the Constitutional Tribunal from December 2015, promoted by the PiS party, was unconstitutional. Also the judgment of 11 August 2016 stated that the new law on the Criminal Code of 22 July 2016 was partly unconstitutional.

37 See: Wyrzykowski (2019), pp. 417–420.

38 See, the Constitutional Tribunal judgments of 3 December 2015, K 34/15, and of 9 December 2015, K 35/15.

39 See, statement of Morawski Lech at the University of Oxford: https://www.youtube.com/watch?v=3p5egncsjm4.

40 See, http://konstytucyjny.pl/esperci-fundacji-batorego-bezpodstawne-zawieszenie-sedziow-tk-w-orzekaniu/.

41 With there being doubts as to the lawfulness of her election.

42 https://www.iustitia.pl/2229-stanowisko-iustitii-w-sprawie-dzialan-rzecznika-praw-obywatelskich-dr-adama-bodnara.

43 Markiewicz (2018), pp. 26–27; Wyrzykowski (2019), p. 418.

44 Quoted in Wojciech Sadurski, How Democracy Dies (in Poland): A case study of Anti-constitutional populist backsliding. p. 30.

45 Wyrzykowski, (2019), p. 418.

46 Act of 8 December 2017 on the Supreme Court (Dz. U. of 2018, item 5).

47 Act of 8 December 2017 amending the Act on the National Council of the Judiciary and some other acts (Dz.U. of 2018, item 3).

48 I have based the above comments on the position of the “Iustitia” Association of Polish Judges: https://www.iustitia.pl/strefa-wewnetrzna/strefa-wiedzy/opinie/1942-opinia-stowarzyszenia-iustitia-o-prezydenckim-projekcie-ustawy-o-sadzie-najwyzszym and https://www.iustitia.pl/strefa-wewnetrzna/strefa-wiedzy/opinie/1939-opinia-stowarzyszenia-iustitia-o-prezydenckim-projekcie-ustawy-o-zmianie-ustawy-o-krajowej-radzie-sadownictwa-oraz-niektorych-innych-ustaw.

49 See: Markiewicz (2018), pp. 28–30.

50 See, the Final Opinion of the ODIHR on the bill on the amendment to the Act on the National Council of the Judiciary and some other acts (http://www.iustitia.pl/images/pliki/KRS/opiniaOBWEkonc.pdf) as well as the *Opinion of Amnesty International on the threat to the independence of courts and impartiality of judges.* It is stressed in both of the opinions that the proposed changes are in violation of the international and regional recommendations in which it is stated that member of councils of the judiciary should be elected by the judiciary.

51 The Act on the organisation of the common courts in the amended wording introduced by means of the Act of 12.7.2017 Dz.U. z 2017 r. poz. 1452 zm; hereinafter Law on the organisation of the common courts.

52 The Constitutional Tribunal clearly concluded that the Minister of Justice, as the administrator of all courts, must undoubtedly have an important impact also on the process of appointing court presidents; however, his voice cannot be the dominant one (Cf. the Constitutional Tribunal ruling of 9 November 1993 in case K 11/93, and the judgment of 18 February 2004 in case K 12/03).

53 The list is available at: https://www.iustitia.pl/informacja-publiczna/2223-decyzje-kadrowe-w-sadach-ad-2018-ujawniamy-pelne-zestawienie.

54 There are district, regional and appellate courts as well as the Supreme Court. For example, a candidate for the KRS, a judge at a District Court in Jarosław, Rafał Puchalski, became president of the Regional Court in Rzeszów after returning from the Ministry of Justice, and has been delegated to the Court of Appeal in Rzeszów.

55 See: Statement by 50 Supreme Court judges: https://www.iustitia.pl/en/new-krs/3849-statement-by-50-supreme-court-judges. See also: The position of Prof. Włodzimierz Wróbel, judge of the Criminal Chamber of the Supreme Court, regarding the obstruction of the SC Assembly on June 12, 2020, by persons appointed with the participation of the neo-NCJ to act as judges of the SC: http://themis-sedziowie.eu/materials-in-english/the-position-of-prof-wlodzimierz-wrobel-judge-of-the-criminal-chamber-of-the-supreme-court-regarding-the-obstruction-of-the-sc-assembly-on-june-12-2020-by-persons-appointed-with-the-participation/.

56 S. 112 § 3 Law on the Common Court System.

57 S. 112 § 6 – 13 Law on the Common Court System.

58 See: https://euobserver.com/justice/145685.

59 For a discussion of the status of the Disciplinary Chamber see W. Wróbel, Izba dyscyplinarna jako sąd wyjątkowy w rozumieniu art. 175 ust. 2 konstytucji RP, Palestra 2019, no 1 - 2, p. 33, on-line versionhttps://palestra.pl/pl/czasopismo/wydanie/1-2-2019/artykul/izba-dyscyplinarna-jako-sad-wyjatkowy-w-rozumieniu-art.-175-ust.-2-konstytucji-rp, http://themis-sedziowie.eu/wp-content/uploads/2020/01/Włodzimierz-Wróbel_Disciplinary-Chamber-as-exceptional-court_def.pdf.

60 This refers to the situation of a woman being interrogated during the 9th month of her pregnancy. In 2013, 6 years after a complaint had been lodged with the CJEU, the government of the Republic of Poland found that there was no possibility of winning the case and offered a settlement. It confirmed that Art. 3 of the European Convention on Human Rights prohibiting torture had been breached with respect to the accountant in question.

61 http://www.krs.pl/pl/rzecznik-prasowy/komunikaty/p,1/6158,komunikat-prasowy-przewodniczacego-krajowej-rady-sadownictwa.

62 See CJEU judgement of 27.02.2018 in the case of C – 64/16 Associação Sindical dos Juízes Portugueses.

63 See the judgement of the ECHR dated 12.03.2019 no. 26374/18, lex no. 2630344.

64 On 8 April 2020 the CJEU ruled that the Disciplinary Chamber of the Polish Supreme Court may not conduct disciplinary proceedings against judges or refer them to courts which do not meet the criterion of independence under Union law. The ruling is in force until the CJEU renders final judgment on the European Commission's complaint against the Polish government. It means that Poland must immediately suspend the application of the national provisions on the powers of the Disciplinary Chamber of the Supreme Court with regard to disciplinary cases concerning judges (Order of the CJEU on 8 April 2020 in case C-791/19 R Commission v Poland, ECLI:EU:C:2020:277).

65 Kurczewski (2016), pp. 181–203.

66 See: Report: Justice under pressure – repressions as a means of attempting to take control over the judiciary and the prosecution in Poland. Years 2015–2019, Ed. J. Kościerzyński, Warsaw 2020, https://www.iustitia.pl/en/activity/informations/3724-report-justice-under-pressure-years-2015-2019.

67 See: Justice under pressure – repressions as a means of attempting to take control over the judiciary and prosecution in Poland. Years 2015–2019, ed. J. Kościerzyński, Warsaw 2020, pp. 36–38, https://www.iustitia.pl/images/pliki/raport2020/Raport_EN.pdf.

68 Case no.: IX Ca 1302/19.

69 Monitor Polski Item 276.

70 First, in a TV programme the Secretary of State at the Justice Ministry, Michał Wójcik, started to publicly threaten Paweł Juszczyszyn with disciplinary proceedings. Then the Justice Minister immediately dismissed Judge Paweł Juszczyszyn from the delegation to the Regional Court without any substantive reasoning.

71 See: Communiqué of the Disciplinary prosecutor of common court judges Piotr Schab on initiating disciplinary proceedings against Judge Paweł Juszczyszyn; judgment of the Supreme Court of 5.12.2019 issued in case III PO 7/18; judgment of the CJEU of 19.11.2019 in joined cases.

72 See: Letter from the deputy disciplinary officer for Common Court Judges Michał Lasota of 9.8.2018, RDSP 712-2/18 calling for a written statement within 14 days; Letter from the deputy disciplinary officer for common court judges Judge Michał Lasota dated 14.8.2018, RDSP 712-3/18 calling for a written statement within 14 days, letter from the deputy disciplinary prosecutor for judges of the common courts, Judge Przemysław W. Radzik of 5.9.2018, RDSP 714-61/18; legal opinion of Prof. Katarzyna Dudka on the scope of application of Article 114 § 2 of the Act of 27.7.2001. – Law on the common courts system (t. one: Journal of Laws of 2018, item 23); letter from the deputy disciplinary officer for judges of the common courts, Judge Przemysław W. Radzik, 8.10.2018, RDSP 712-12/18, calling for a written statement within 14 days; letter from the deputy disciplinary officer for judges of the common courts, Judge Przemysław W. Radzik, 8.10.2018, RDSP 712-13/18, calling for a written statement within 14 days.

73 See: Letter from the deputy disciplinary prosecutor for judges of the common courts, Judge Przemysław W. Radzik, 8.10.2018, RDSP 712-13/18; The decision of the deputy disciplinary prosecutor for judges of the common courts, judge Michał Lasota, of 22.11.2018 issued in the case of RDSP 712-14/18 on the initiation of disciplinary proceedings against the judge of the District Court Waldemar Żurek and the presentation of charges for disciplinary offences.

74 See: Report: Justice under pressure – repressions as a means of attempting to take control over the judiciary and the prosecution in Poland. Years 2015–2019, Ed. J. Kościerzyński, Warsaw 2020, p. 73, https://www.iustitia.pl/en/activity/informations/3724-report-justice-under-pressure-years-2015-2019.

75 See: Announcements by the Disciplinary Officer of the General Court Judges Piotr Schab on 15 and 18 December 2019 regarding the initiation of disciplinary proceedings against judges Aleksandra Janas and Irena Piotrowska; Supreme Court judgment of 5 December 2019 issued in case III PO 7/18; judgment of the CJEU of 19 November 2019 in joined cases AK (C-585/18), CP (C-624/18) and DO (C-625/18).

76 See. Lenaerts (2020), p. 30.

77 As in: judgement dated 16 July 2015, CEZ Razpredelenie Byłgarija, C-83/14, EU:C:2015:480, point 71 and the case law quoted therein.

78 See also the judgement dated 31 January 2013, D. and A., C-175/11, EU:C:2013:45, point  99; and also the decisions of the ECHR dated 28  June 1984 in Campbell and Fell v. the United Kingdom, CE:ECHR:1984:0628JUD000781977, § 79; dated 2 June 2005 in the case of Zolotas v. Greece, CE:ECHR:2005:0602JUD003824002, §§ 24, 25; dated 9 November 2006 in the case of Sacilor Lormines v. France, CE:ECHR:2006:1109JUD006541101, § 67; and also dated 18 October 2018 in the case of Thiam versus France, CE:ECHR:2018:1018JUD008001812, § 80 and the case law quoted therein.

79 In this respect it should be reiterated that in the judgement dated 24 June 2019 Commission/Poland (Independence of the Supreme Court)(C 619/18, EU:C:2019:531), the CJ found that due to the adoption of the said measures the Republic of Poland had breached the irremovability and independence of the judges of the Supreme Court and had failed to perform its obligations under Art. 19(1)(2nd par.) TEU.

80 Case file no.: III PO 7/18.

81 III PO7/18, I NOZP 3/19 (7), II DSI 17/18, III KO154/18, III KZP 4/19.

82 The resolution of the combined Civil, Criminal and Labour and Social Insurance Chambers of the Supreme Court dated 23 January 2020, Case BSA I-4110-1/20, publ. http://www.sn.pl/aktualnosci/SiteAssets/Lists/Wydarzenia/AllItems/BSA%20I-4110-1_20_English.pdf.

83 It should be noted that in recognizing this resolution on 24 January 2020 the Supreme Court, having already heard the legal issue in the Civil Chamber in case file no. III CZP 94/19, refused to adopt a resolution on the answer to the following legal issue: “Does the participation of the National Council of the Judiciary whose composition was formed as a result of the election by the Polish Sejm of fifteen judges in the manner described by the provisions of the Act dated 8 December 2017 on the Change to the Act on the National Council of the Judiciary and Certain Other Acts (Dz.U. of 2018 item 3 as amended) in the process of appointing a judge being a member of the adjudicating panel constitute an autonomous and sufficient condition to find that the composition of the court is contrary to the provisions of law as defined in s. 379(4) of the CivPC?” The Supreme Court indicated that on 23 January 2020 a resolution by the combined Civil, Criminal and Labour and Social Insurance Chambers of the Supreme Court, court file no. BSA 1-4110-1/20, had been adopted. This resolution has the effect of a legal principle that is binding on all the adjudicating panels of the Supreme Court and in its second point it settles, in a binding way, the legal issue presented in this case, which in the light of the case law of the Supreme Court leads to the necessity to refuse to adopt the resolution.

84 The resolution of the combined Civil, Criminal and Labour and Social Insurance Chambers of the Supreme Court dated 23 January 2020, Case BSA I-4110-1/20, point 12 et seq., http://www.sn.pl/aktualnosci/SiteAssets/Lists/Wydarzenia/AllItems/BSA%20I-4110-1_20_English.pdf.

85 http://www.sn.pl/aktualnosci/SitePages/Wydarzenia.aspx?ItemSID = 601-0dc69815-3ade-42fa-bbb8-549c3c6969c5&ListName = Wydarzenia.

86 E. Łętowska, A wise, forward-looking resolution by the Supreme, Court: https://ruleoflaw.pl/letowska-a-wise-forward-looking-resolution-by-the-supreme-court/.

87 W. Wróbel, The Disciplinary Chamber of the Supreme Court as an exceptional court in the meaning of Article 175, para. 2 of the Polish Constitution (original title: “Izba dyscyplinarna jako sąd wyjątkowy w świetle Article 175 ust. 2 Konstytucji RP”, “Palestra” No. 1-2/2019, an academic and socio-cultural magazine published by the Polish Supreme Bar Council, the article can be accessed at: http://czasopismo.palestra.pl/upload/15/54/10/1554104640_.pdf, accessed on 05/04/2019), p. 33, on-line version: http://themis-sedziowie.eu/wp-content/uploads/2020/01/Włodzimierz-Wróbel_Disciplinary-Chamber-as-exceptional-court_def.pdf.

88 W. Wróbel, The Disciplinary Chamber of the Supreme Court as an exceptional court in the meaning of Article 175, para. 2 of the Polish Constitution (original title: “Izba dyscyplinarna jako sąd wyjątkowy w świetle Article 175 ust. 2 Konstytucji RP”, “Palestra” No. 1-2/2019, an academic and socio-cultural magazine published by the Polish Supreme Bar Council, this article can be accessed at: http://czasopismo.palestra.pl/upload/15/54/10/1554104640_.pdf, accessed on 05/04/2019), p. 33, on-line version: http://themis-sedziowie.eu/wp-content/uploads/2020/01/Włodzimierz-Wróbel_Disciplinary-Chamber-as-exceptional-court_def.pdf.

89 See: Kryger (2016), pp. 199–229; Krygier (2019), pp. 106–136.

90 Kurczewski (2016), pp. 181–203. See also: J. Kurczewski, “Rządy prawa” [The Rule of Law] in Res Publica, 3 (March) 1989. Kurczewski (1994); Kurczewski (1995).

91 Kurczewski (2016), pp. 181–203.

92 See: Kurczewski (1993).

93 Markowski (2019), pp. 111–132.

94 Kurczewski (2016), pp. 181–203.

95 The National Prosecutor’s Office wants to waive the immunity of judge Igor Tuleya in order to bring criminal charges against him. The matter concerns his alleged failure to perform his official duties and exceeding his rights in connection with a judgement issued on 18 December 2017. According to the National Prosecutor’s Office, Tuleya unlawfully allowed journalists to hear and record him. See: https://ruleoflaw.pl/the-disciplinary-chamber-will-examine-the-case-of-judge-igor-tuleya-despite-cjeu-order/.

96 See: I. Tuleya, Poland ‘crossed rubicon’ against EU court injunction: https://euobserver.com/justice/148609.
